# Flavonoids Isolated from Korea *Citrus aurantium* L. Induce G2/M Phase Arrest and Apoptosis in Human Gastric Cancer AGS Cells

**DOI:** 10.1155/2012/515901

**Published:** 2011-12-13

**Authors:** Do-Hoon Lee, Kwang-Il Park, Hyeon-Soo Park, Sang-Rim Kang, Arulkumar Nagappan, Jin-A Kim, Eun-Hee Kim, Won-Sup Lee, Young-Sool Hah, Hyon-Jong Chung, Su-Jin An, Gon-Sup Kim

**Affiliations:** ^1^Research Institute of Life science and College of Veterinary Medicine, Gyeongsang National University, Gazwa, Jinju 660-701, Republic of Korea; ^2^Korea National Animal Research Resource Center and Korea National Animal Bio-resource Bank, Gyeongsang National University, Gazwa, Jinju 660-701, Republic of Korea; ^3^Department of Nursing Science, International University of Korea, Jinju 660-759, Republic of Korea; ^4^Department of Internal Medicine, Institute of Health Sciences, Gyeongsang National University School of Medicine and Gyeongnam Regional Cancer Center, Gyeongsang National University Hospital, Jinju 660-702, Republic of Korea; ^5^Clinical Research Institute, Gyeongsang National University Hospital, Jinju 660-702, Republic of Korea

## Abstract

*Aim of the Study*. Citrus species is used in traditional medicine as medicinal herb in several Asian countries including Korea. Flavonioids became known as various properties, such as anti-oxidants, anti-inflammation and anti-cancer, and so forth. The present study, the anti-cancer effect of flavonioids isolated from *Citrus aurantium* L. in human gastric cancer AGS cells has been investigated. *Materials and Methods*. The anti-proliferative activity was assayed using MTT assay. Cell cycle analysis was done using flow cytometry and apoptosis detection was done using by hoechst fluorescent staining and Annexin V-propidium iodide double staining. Western blot was used to detect the expression of protein related with cell cycle and apoptosis. *Results*. Flavonoids isolated from *Citrus aurantium* L. have the effect of anti proliferation on AGS cells with IC50 value of 99 **μ**g/mL. Flavonoids inhibited cell cycle progression in the G2/M phase and decrease expression level of cyclin B1, cdc 2, cdc 25c. Flavonoids induced apoptosis through activate caspase and inactivate PARP. *Conclusions*. Flavonoids isolated from *Citrus aurantium* L. induced G2/M phase arrest through the modulation of cell cycle related proteins and apoptosis through activation caspase. These finding suggest flavonoids isolated from *Citrus aurantium* L. were useful agent for the chemoprevention of gastric cancer.

## 1. Introduction

Citrus fruits are a very popular food source because of their nutrient, flavor, and intrinsic attributes [1] and have long been the basis of commonly used traditional medicines in several Asian countries. Immature peels of citrus fruit are used to treat indigestion and have demonstrated potential as a chemotherapeutic agent [2, 3]. Among nutrients of citrus fruits, flavonoids have been more recently recognized as having various medicinal benefits that include antioxidant, antimicrobial, anti-inflammatory, and anticancer activities [4, 5]. Citrus fruits have numerous bioactive flavonoids such as naringin, naringenin, narirutin, nobiletin, quercetin, kaempferol, hesperidin, neohesperidin, didymin, and poncirin [6]. In particular, naringin, nobiletin, and hesperidin display anticancer effects via cell cycle arrest and apoptosis [7–9].

Gastric cancer is the most prevalent cancer in Korea. According to Ministry of Health and Welfare statistics, there were 28,078 cases of gastric cancer in Korea in 2008, representing 15.7% of all cancer cases in the country. Globally, gastric cancer is the fourth leading cancer and is the second-leading cause of cancer-related death, following lung cancer [10]. Treatment of gastric cancer consists generally of surgery, chemotherapy, and/or radiotherapy. While the treatments can be effective, about half of gastric cancer patients are untreatable [11]. The need for more effective treatments is pressing.

The programmed cell death mechanism of apoptosis is the major regulator of cell proliferation and is a focus of cancer research as an effective way of eliminating precancerous and/or cancerous cells [1]. Caspases play a pivotal role in apoptosis; their overexpression and cleavage is a precursor of apoptosis in mammalian cells. To date, 14 caspases have been identified based on their function. They constitute three functional groups: inflammatory caspases (caspase-1, -4, and -5), apoptotic initiator caspases (caspase-2, -8, -9, and -10), and apoptotic effector caspases (caspase-3, -6, and -7) [12]. Caspases possess inactive zymogens that consist of p20 (large) and p10 (small) subunits. In response to apoptotic signals, the inactive zymogens are cleaved, yielding active forms of the proteins that are associated with the induction of apoptosis [13]. Also, the Bcl-2 family of proteins, whose activity is directed to act at the mitochondrial outer membrane, are major regulators of apoptosis. The Bcl-2 family consists of proapoptotic and antiapoptotic members [14]. Apoptotic Bcl-2 proteins such as Bax and Bak created pores in the mitochondrial outer membrane, through which cytochrome c is released to the cytosol. Binding of cytochrome c to Apaf-1 creates an apoptosome complex that activates caspase-9, which in turn activates caspase-3. Antiapoptotic Bcl-2 proteins such as Bcl-2 and Bcl-xL preserve the mitochondrial membrane structure through interaction with apoptotic Bcl-2 proteins [15].

 Cell cycle arrest is also a major regulator of cell proliferation. In eukaryotic cells, the cell cycle comprises the G1, S, G2, and M phases. Checkpoints to the cycle are present in the G1 and G2 phases. In cell cycle progression, cyclins and cyclin-dependent kinases (cdks) play a central role as regulators. In the mid-G1 phase, cell cycle progression is controlled by a cyclin D-cdk4/cdk6 complex. In late-G1, progression is controlled by the cyclin E-cdk2 complex. In the G2 phase, cell cycle progression is controlled by the cyclin A/B-cdc2 complex [16–18]. DNA damage can inhibit cell proliferation by the inactivation of cyclins and cdks and subsequent cell cycle arrest.

As referred to earlier, flavonoids isolated from *Citrus aurantium* L. have the anticancer effect. However, the mechanisms of the anticancer activity of Korea *Citrus aurantium* L. still remain unknown. In the present study, we demonstrate that flavonoids isolated from Korea *Citrus aurantium* L. cause cell cycle arrest and apoptosis in AGS human gastric cancer cells. The expression levels of several important proteins are shown to be strongly related with the cell cycle and apoptosis. Finally, the occurrence of cell cycle arrest and apoptosis was determined to ascertain the anticancer mechanism of the isolated flavonoids.

## 2. Materials and Methods

### 2.1. Antibodies and Reagents

Cyclin B1, cdc 2, cdc 25c, and ß-actin were purchased from Millipore (Billerica, MA, USA). Antibodies for Bcl-xL, Bax, cleaved poly(ADP-ribose) polymerase (PARP), and caspases-3, -6, -8, and -9 were purchased from Cell Signaling Technology (Danvers, Mass, USA). Horseradish peroxidase- (HRP-) coupled goat anti-mouse IgG and anti-rabbit IgG were purchased from Santa Cruz Biotechnology (Santa Cruz, CA, USA). RPMI-1640 was purchased from Hyclone (Logan, UT, USA). Fetal bovine serum (FBS) and antibiotics (streptomycin/penicillin) were purchased from Gibco (BRL Life Technologies, Grand Island, NY, USA). 3-(4,5-Dimethylthiazol-2-yl)-2,5-dephenyltetrazolium bromide (MTT), dimethylsulfoxide (DMSO), and RNase A were obtained from Sigma-Aldrich (St. Louis, MO, USA). Fluorescein isothiocyanate (FITC) annexin-V apoptosis detection kit 1 was purchased from BD Pharmingen (San Diego, CA, USA). Enhanced chemiluminescence (ECL) kit was purchased from Amersham Life Science (Buckinghamshire, UK). Materials and chemicals used for electrophoresis were obtained from Bio-Rad Laboratories (Hercules. CA, USA).

### 2.2. Isolation of Flavonoids

**Figure 1:**
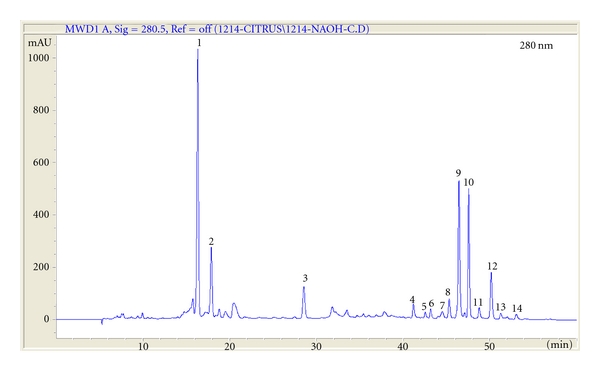
HPLC chromatogram patterns of Korea *Citrus aurantium* L. at 280 nm. (1) naringin, (2) hesperidin, (3) poncirin, (4) isosiennsetin, (5) hexamethoxyflavone, (6) sineesytin, (7) hexamethoxyflavone, (8) tetramrthnl-o-isoscutellaeein, (9) nobiletin, (10) heptamethoxyflavone, (11) 3-hydoxynobiletin, (12) tangeretin, (13) hydroxypentamethoxyflavone, and (14) hexamethoxyflavone.

The analyses of isolation of flavonoids were conducted at the Department of Chemistry, Gyeongsang National University by Professor Sung Chul Shin. High-performance liquid chromatography (HPLC) was performed as descried. Briefly, HPLC analysis was performed using a 1100 series LC system installed with a G1322A degasser, a G1312A pump, a G1313A autosampler, and a G1316A oven (Agilent Technologies, Palo Alto, CA, USA). Chromatographic separation was carried out on a Zorbax StableBond Analytical SB-C18 column (4.6 × 250 mm, 5 *μ*m; Agilent Technologies). The binary solvent system was made up of 0.1% aqueous formic acid (A) and methanol/acetonitrile (1 : 1) (B). Elution was performed using a linear gradient (0%–25% B) over 10 min and 40%–70% B over 10 min, followed by 30 min of isocratic elution, declined from 40%–25% B over 5 min and followed by 10 min of isocratic elution. The flow rate was 0.5 mL/min in column temperature of 35°C and an infusion volume of 10 *μ*L in each experiment. Chromatographic data were collected and controlled using a ChemStation, Rev.B.0301 (Agilent Technologies). Spectral data were collected (200–400 nm, 2 nm resolution) for the entire progression, and the flavonoids were quantified by extracting each chromatogram at 280 nm (Figure 1). All flavanones and flavones were quantified using the external calibration curves of hesperetin and nobiletin, respectively. Tandem mass spectrometry (MS/MS) experiments were conducted using a 3200 QTRAP LC-MS/MS system (Applied Biosystems, Foster City, CA, USA) with a Turbo V-source and a Turbo Ion Spray probe (Applied Biosystems). The mass spectrometer was operated in the positive mode with selected ion monitoring (SIM), BioAnalyst, version 1.4.2 (AB Sciex, Zagreb, Croatia). The electron spray voltage was set at 5.2 kV and the source temperature at 500°C. The mass spectra were recorded between m/z 100 and m/z 1000 with a step size of 0.06 amu.

### 2.3. Cell Culture

AGS human gastric cancer cells obtained from the Korea Cell Line Bank (Seoul, Korea) were cultured in RPMI-1640 medium supplemented with 10% heat-inactivated fetal calf serum and 100 *μ*g/mL of penicillin and streptomycin in a humidified atmosphere of 5% CO_2_ at 37°C.

### 2.4. Cell Viability Assay and Morphological Examination

Cell viability was determined using MTT 24 h after the experimental treatments. Briefly, cells were plated in wells of 12-well plates and incubated for 24 h at 37°C. The cells were untreated or treated with varying concentrations of flavonoids (10, 50, 100, 150, and 200 *μ*g/mL) for 24 h at 37°C. DMSO (0.1%) was used as a vehicle control. MTT solution (5 mg/mL in phosphate buffered saline; PBS) was diluted to 0.5 mg/mL by medium. After 3 h incubation at 37°C, MTT-containing medium was removed and the crystals that had formed were dissolved by the addition of DMSO to each well. After mixing, the absorbance of the cells was measured at 540 nm by using an enzyme-linked immunosorbent assay plate reader. For morphological examination, cells were grown on 6-well plates, treated with flavonoids for 24 h, and then examined under light microscopy (×400).

### 2.5. Hoechst 33258 Fluorescent Staining

AGS cells from exponentially growing cultures were seeded in 12-well culture plates. AGS cells were untreated or treated with varying concentrations of flavonoids (20, 40, 60, 80, and 100 *μ*g/mL) for 24 h at 37°C. The cells were then washed in ice-cold phosphate-buffered saline (PBS) and fixed in a solution of 3.7% paraformaldehyde for 15 min at room temperature. To identify the apoptotic AGS cells, they were stained with Hoechst 33258 (5 *μ*g/mL in PBS) for 10 min at room temperature. The nuclei structure of the cells was examined by Leica fluorescence microscopy. The apoptotic cells were observed at 1000x magnification.

### 2.6. Cell Cycle Analysis

Upon reaching 70%–80% confluence, AGS cells were untreated or treated with varying concentrations of flavonoids (20, 40, 60, 80, and 100 *μ*g/mL) for 24 h at 37°C. Then, the cells were washed twice with cold PBS, trypsinized, and centrifuged. The cells were fixed in 70% (v/v) ethanol for 24 h at 4°C. The cells were washed with PBS and stained with propidium iodide (PI; 50 *μ*g/mL) including RNase A (0.1 mg/mL) in PBS (pH 7.4) for 30 min in the dark. PI-stained samples were analyzed with a FACScan flow cytometer (Becton Dickinson, San Jose, CA, USA). In each sample, 10,000 cells were sorted. The data were analyzed using CellQuest software (Becton Dickinson).

### 2.7. Apoptosis Assays

Apoptotic cells were detected using a FITC annexin-V apoptosis detection kit 1 (BD Pharmingen, San Diego, CA, USA). Briefly, cells were untreated or treated with varying concentrations of flavonoids (20, 40, 60, 80, and 100 *μ*g/mL) for 24 h at 37°C. The cells were trypsinized and washed with PBS. The washed cells were resuspended in Annexin-V binding buffer containing 10 mM HEPES/NaOH, pH 7.4, 140 mM NaCl and 2.5 mM CaCl2 according to the manufacturer's protocol. The cells were stained simultaneously with FITC-conjugated Annexin-V and PI at room temperature for 15 min in the dark, prior to the addition of binding buffer. The apoptotic cells were measured using a FACScan flow cytometer. The cells were sorted into intact cells (Annexin V− PI−), early apoptotic cells (Annexin V+ PI−), late apoptotic cells (Annexin V+ PI+), and necrotic cells (Annexin V− PI+).

### 2.8. Caspase-3 Activity

Caspase-3-activity was determined by a colorimetric assay using a caspase-3 activation kit according to the protocol of the manufacturer (Millipore, Billerica, MA, USA). In brief, the cells were lysed in the supplied lysis buffer and incubated for 10 min in an ice bath. Then, the samples were centrifuged for 5 minutes in a microcentrifuge (10,000 ×g). The supernatants were collected and incubated with Assay buffer and Caspase-3 Substrate (Ac-DEVD-pNA) for 1 h at 37°C. The optical density of the reaction mixture was quantified using an enzyme-linked immunosorbent assay plate reader at 405 nm.

### 2.9. Western Blot Analysis

Protein concentration was determined using a Bradford assay (Bio-Rad). An equal protein amount was separated by 12% sodium dodecyl sulfate-polyacrylamide gel electrophoresis (SDS-PAGE), and the resolved proteins were transferred onto 0.45 mm Immobilon-P polyvinylidene fluoride (PVDF) membranes (Millipore). Each membrane was blocked with TBST (10 mM Tris-HCl, pH 7.4, 150 mM NaCl, 0.1% Tween-20) containing 5% skin milk. Blocking was performed for 1 h at room temperature. Then, each membrane was incubated with primary antibody (1 : 1000 dilution) for overnight at 4°C, washed five times for 10 minutes each time with TBST, and incubated with HRP-conjugated secondary antibody (1 : 2000 dilution) for 2 h at room temperature. Each membrane was washed 5 times for 10 minutes each time with TBST. Protein bands were visualized by ECL and Western Blotting Detection Reagents and exposed to X-ray films (Fuji, Tokyo, Japan). Each band was analyzed using the Image J program (http://rsb.info.nih.gov/).

### 2.10. Statistical Analysis

All experiments were performed in triplicate. Results are expressed as the mean ± standard deviation of at least three separate experiments. Statistical analysis was determined by Student's *t*-test using SPSS version 10.0 for Window (SPSS, Chicago, IL, USA). A value of *P* < 0.05 was considered to be significant.

## 3. Results

### 3.1. Flavonoids Inhibit Cell Viability

**Figure 2:**
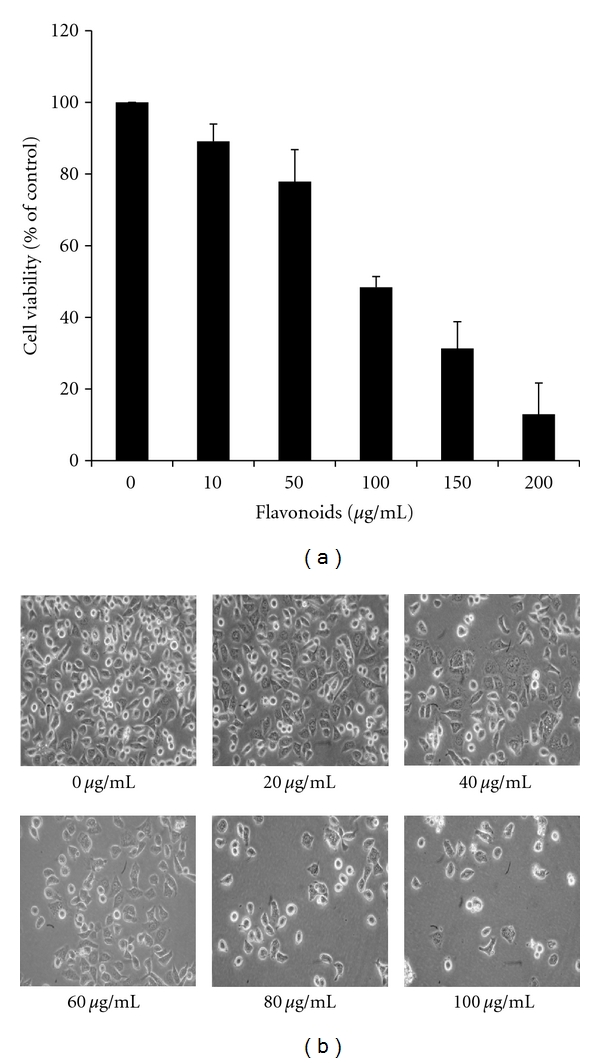
Effect of flavonoids on the viability of AGS cells. (a) AGS cells were treated with various concentrations of the flavonoids for 24 h. Cell viability was then determined by an MTT assay. Cell viability is represented as the percentage relative absorbance compared to the controls. (b) Morphology of cells treated with or without flavonoids for 24 h and examined under light microscopy (×400). Each value represents the mean of three experiments.

In order to estimate the effect of cell viability inhibition, we tested the cytotoxicity of various concentrations (0–200 *μ*g/mL) of flavonoids for 24 using the MTT assay. As shown in Figure 2, flavonoids inhibited cell viability in a dose-dependent manner. Compared with the control group, the viability of cells treated with flavonoid concentrations of 100 *μ*g/mL declined by about 48% after 24 h (IC50 approximately 99 *μ*g/mL). Visual observations revealed a dose-dependent change in cell shape such as cell shrinkage.

### 3.2. Flavonoids Induce G2/M Phase Cell Cycle Arrest

As shown in Figure 4, flavonoids arrested the cell cycle in a dose-dependent fashion at the G2/M phase. The S phase was significantly decreased in AGS cells treated with flavonoid concentrations of 60, 80, and 100 *μ*g/mL. Comparison of control cells and cells treated with 100 *μ*g/mL of flavonoids for 24 h revealed an increase in the G2/M phase from 34.29% to 49.23% (*P* < 0.01) and a decrease in the S phase from 29.54% to 18.95% (*P* < 0.01). But, flavonoids did not influence the G0/G1 phase. These data indicated that flavonoids caused G2/M phase arrest in AGS cells.

### 3.3. Flavonoids Inhibit Expression of Cyclin B1, cdc 2, and cdc 25c

Cyclin B1, cdc 2, and cdc 25c are important proteins related to the G2/M phase. G2/M phase was controlled by a complex formed cyclin B1 and cdc 2; the complex is regulated by cdc 25c. Because flavonoids induce G2/M phase arrest in AGS cells, we evaluated the expression of proteins that regulate the G2/M phase transition using Western blot. As shown in Figure 4, the protein levels of cyclin B1, cdc 2, and cdc 25c decreased in a dose-dependent manner, with significant inhibition occurring at flavonoid concentrations of 80 and 100 *μ*g/mL. These data indicated that flavonoids reduce the expression of cyclin B1, cdc 2, and cdc 25c.

### 3.4. Flavonoids Induce Apoptosis

Apoptosis is another reason of inhibited cell proliferation. The induction of apoptosis by flavonoid exposure was presently assessed using Hoechst staining and Annexin V-PI double staining. Treatment with 100 *μ*g/mL flavonoids for 24 h in AGS cells produced intense Hoechst-positive staining for condensed nuclei indicative of apoptosis (Figure 5(a)). Also treatment with 0, 20, 40, 60, 80, and 100 *μ*g/mL of the flavonoids for 24 h progressively decreased the proportion of intact cells (Figure 5(b)), coincident with a dose-dependent increase in apoptosis. Comparison of control group cells and cells treated with 100 *μ*g/mL of the tested flavonoids for 24 h revealed a decrease in prevalence of intact cells from 88.26% to 42.42% (*P* < 0.01). In contrast, early apoptotic cells increased from 10.29% to 48.07% (*P* < 0.01), and late apoptotic cells increased from 1.34% to 9.44% (*P* < 0.05). These data indicated that flavonoids induced apoptosis in AGS cells.

### 3.5. Flavonoids Activate Caspases and Shift the Bax/Bcl-xL Ratio

The observation that the flavonoids induced apoptosis of AGS cells prompted an examination of the expression levels of some apoptosis regulatory proteins, including Bcl-xL, Bax, procaspase-3, -6, -8, and -9, and cleaved PARP. Bcl-xL and Bax are major proteins of the mitochondria apoptosis pathway. Western blot results showed that expression level of Bcl-xL was decreased in a dose-dependent manner, whereas the level of Bax increased. The effect was the significantly increased Bax/Bcl-xL ratio in the presence of flavonoid concentrations of 60, 80, and 100 *μ*g/mL (Figure 7). Caspases are also major proteins of apoptosis. Presently, the levels of procaspase-3, -6,-8, and -9 were diminished in a dose-dependent manner (Figure 6(a)). The observation of diminished procaspase-3 expression prompted an examination of caspase-3 -activity in AGS cells treated with flavonoids. Treatment with the flanovoids significantly increased caspase-3 activity at 60, 80, and 100 *μ*g/mL (Figure 6(g)). Cleavage of PARP is the main hallmark for caspase-dependent apoptosis. Cleaved PARP also increased in a dose-dependent manner. The collective data favored the suggestion that flavonoids induced apoptosis through a shift in Bax/Bcl-xL ratio and the activation of caspases.

## 4. Discussion and Conclusions

Plant-derived herbal medicines have been used for a long time in several Asian countries. Several studies reported anticancer activity in vitro using a natural herb extract [19, 20]. In recent years, interest in flavonoids isolated from medicinal herbs such as *Silybum marianum, Alpinia officinarum, *and* Hypericum perforatum* has been increasing because of their anticancer effects on various cancer cells. Furthermore, natural flavonoids advantageously do not possess any side effects [21, 22]. In this study, the main flavonoids isolated from *Citrus aurantium* L. were naringin, nobiletin, and hesperidin. These components confer superb anticancer effects that appear through cell cycle arrest and apoptosis in various cancers [8, 9, 23] (Table 1).

In the present study, the anticancer effects of flavonoids isolated from *Citrus aurantium* L. on AGS gastric carcinoma cells were investigated. Viability and motility assays demonstrated that flavonoids suppressed proliferation of AGS cells compared with untreated control cells. Regulating the cell cycle and apoptosis is crucial to retaining homeostasis between cell division and cell death [24].

**Figure 3:**
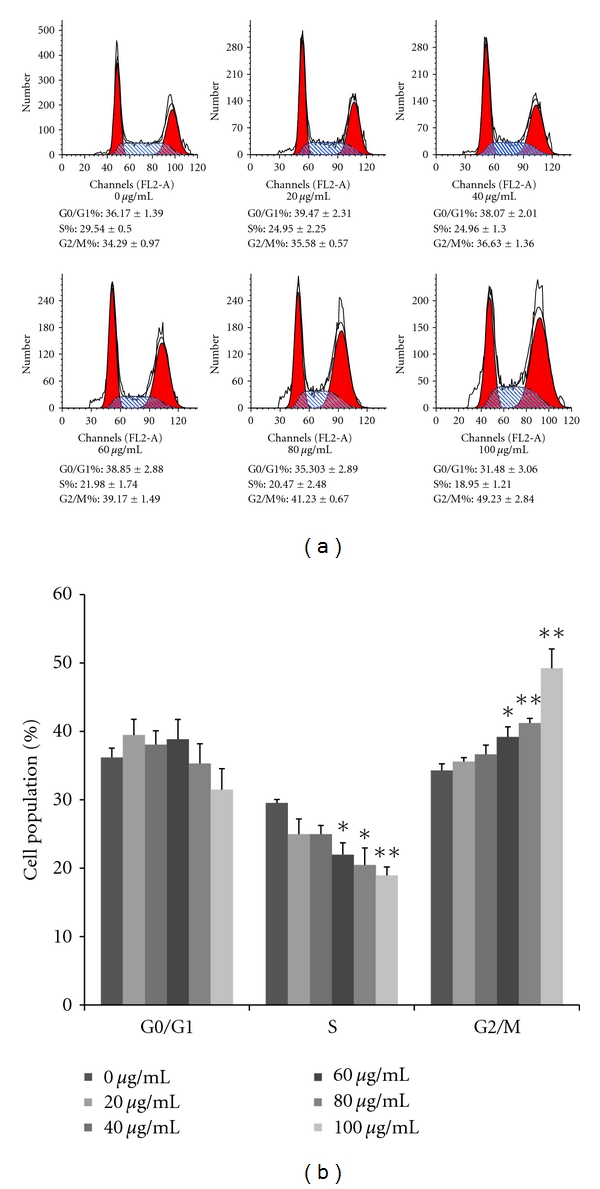
Flavonoids induce G2/M phage arrest on AGS cells. Flavonoids were treated with the indicated concentrations, and cells were incubated for 24 h. Cell cycle proportions were determined by flow cytometry after staining with propidium iodide. (a) Flow cytometry of cell cycle phase distribution. (b) Statistical analysis of cell cycle phase distribution. Each value represents the mean of three experiments; bars, ±SD. (*P < 0.05, **P < 0.01 versus control group.)

**Figure 4:**
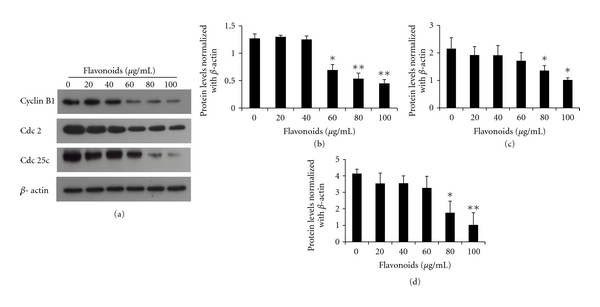
Flavonoids alter the expression of cell-cycle-related proteins cdc 2, cyclin B1, and cdc 25c. The expression of proteins was assessed by immunoblotting. (a) Representative blots are shown. Densitometric analysis of the effect of flavonoids on the expression of cyclin B1 (b), cdc 2 (c), and cdc 25c (d). Each value represents the mean of three experiments; bars, ±SD. (*P < 0.05, **P < 0.01 versus control group.)

**Figure 5:**
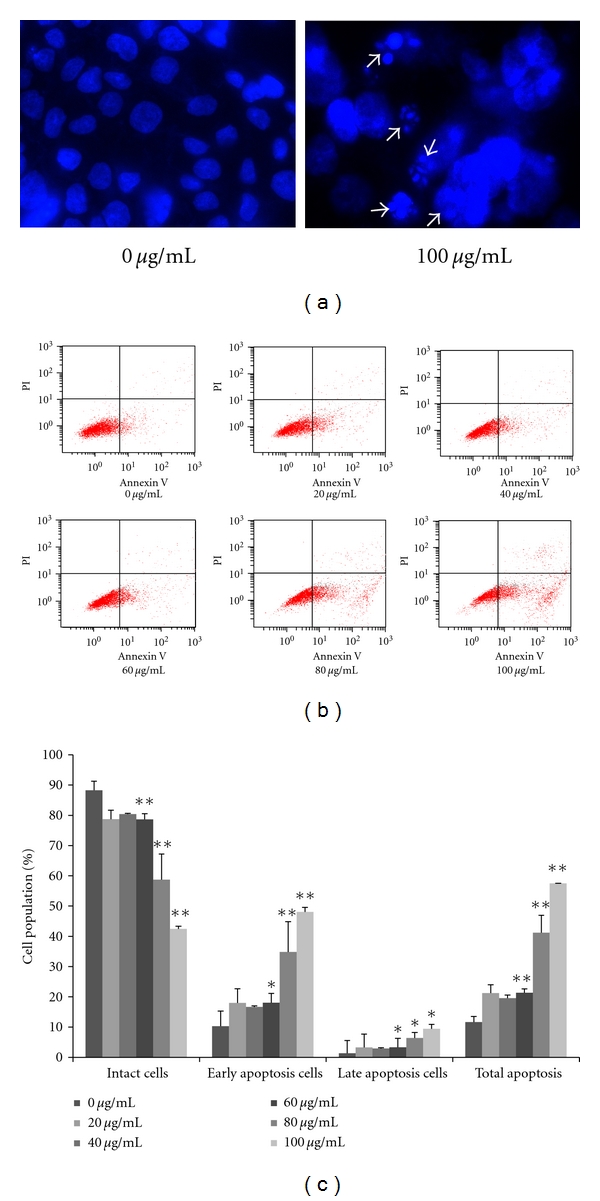
Flavonoids induce apoptosis of AGS cells. Flavonoids were added at the indicated concentrations, and cells were incubated for 24 h. (a) Hoechst 33258 staining of AGS cells treated with or without flavonoids for 24 h. Fragmented or condensed nuclei could be observed at 1000x magnification in the flavonoids-treated group as indicated by the arrows. (b) Apoptosis was assessed by Annexin V-PI double staining. (c) Statistical analysis of apoptosis rate. Each value represents the mean of three experiments; bars, ±SD. (*P < 0.05, **P < 0.01 versus control group.)

**Figure 6:**
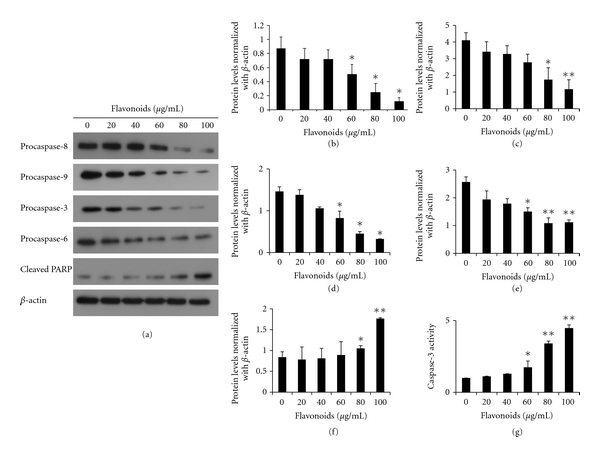
Flavonoids alter the expression of apoptosis-related proteins caspase-8, -9, -3, and -6, and cleavage of PARP. The expression of proteins was assessed by immunoblotting. (a) Representative blots are shown. Densitometric analysis of the effect of flavonoids on the expression of procaspase-8 (b), -9 (c), -3 (d), and -6 (e), and cleaved PARP (f). (g) Caspase-3 activity was determined using a colorimetric caspase-3 activation kit assay kit following the manufacturer's protocol. Each value represents the mean of three experiments; bars, ±SD. (*P < 0.05, **P < 0.01 versus control group.)

**Figure 7:**
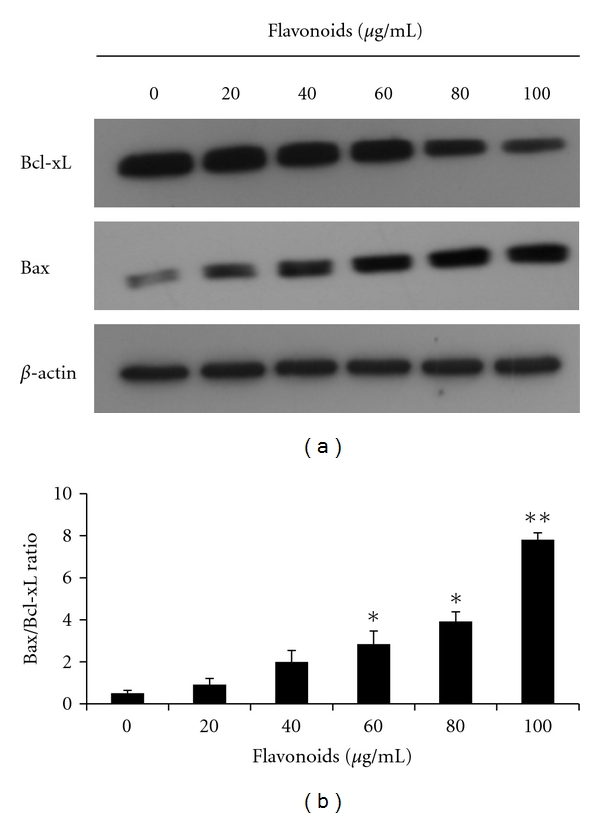
Flavonoids alter the expression of mitochondria-dependent pathway of apoptosis-related proteins Bcl-xL and Bax. The expression of proteins was assessed by immunoblotting. (a) Representative blots are shown. (b) Densitometric analysis of the Bax/Bcl-xL ratio. Each value represents the mean of three experiments; bars, ±SD. (*P < 0.05, **P < 0.01 versus control group.)

Cell cycle aperiodicity is a classic feature of tumor cells [25]. In the cell cycle, the G2/M checkpoint represents a potential target for cancer therapy. When cells that contain damaged DNA enter mitosis, the G2/M checkpoint helps prevent cell cycle progression in an effort to repair of DNA that was damaged in late S or G2 phase [26]. Quercetin suppresses the proliferation of human cervical cancer cells by inducing G2/M phase cell cycle arrest [27]. Also, genistein induces G2/M phase cell cycle arrest in nasopharyngeal carcinoma cells [28]. Presently, AGS cells treated with various concentrations of flavonoids accumulated mostly in the G2/M phase in a dose-dependent manner, whereas the S phase decreased. In terms of regulating the cell cycle, cdcs and cyclins play a most influential role. The complex formed between cyclin B and cdc 2 is crucial in the transition from the G2 to M phase [29]. At the end of the G2 phase, cdc 2 is activated by dephosphorylation on Thr14/Tyr15 and complexes with cyclin B. Cdc 25c is very important in cdc 2 activation. Phosphorylation of cdc 25c plays an important part in irreversible G2/M phase arrest [30]. Presently, the tested flavonoids downregulated cyclin B1, cdc 2, and cdc 25c protein levels, which were required for the processing of the G2/M phase. Flavonoids such as luteolin and licochalcone A suppress the growth of AGS cells by blocking cell cycle progression at the G2/M phases. In these phases, cyclin B, cdc 2, and cdc 25c play critical roles. Similar effects reported that luteolin and licochalcone A induce the downregulation of the production of cyclin B, cdc 2, and cdc 25c [31, 32]. The collective previous and present results support a powerful inhibitory effect of cyclin B, cdc 2, and cdc 25c and provide molecular evidence of the antiproliferative effect directed at G2/M phase arrest. Decrease of cyclin B1, cdc 2, and cdc 25c expression might be a molecular mechanism through which flavonoids induce G2/M arrest (Figure 3).

Apoptosis is programmed cell death that can occur by a variety of internal or external stimuli, and these signals are controlled by two distinct pathways. One is an extrinsic pathway (death receptor pathway), and the other is an intrinsic pathway (mitochondria pathway) [33]. Caspases have a major role in the extrinsic and intrinsic pathways of apoptosis. In the regulation of apoptosis, caspases are divided into two groups: initiator caspases (caspase-2, -8, -9, and -10) and effector caspases (caspase-3, -6, and -7). Initiator caspases are activated as a result of protein complex formation, such as the death-inducing signaling complex (DISC) and apoptosome [24, 34]. The effector caspases-3 and -6 are activated through the extrinsic pathway that involves caspase-8 that involves caspase-9. Caspase-3 is a prominent effector that cleaves the various elements related to apoptosis. Because of similarities in their substrate specificity, caspase-6 is able to partially assume the functional role of caspase-3 [35, 36]. PARP, as a DNA repair enzyme, is activated by DNA breaks and promotes the attachment of ADP-ribose polymers to various nuclear factors, expediting repair. PARP is an important factor for cell viability. But, the cleavage of PARP helps cellular disassembly and is a marker of cells undergoing apoptosis. During apoptosis, caspases induce PARP cleavage and inactivation [37]. Caspase signaling is initiated and proceeds by proteolytic autocatalysis and by cleavage of downstream caspases and substrates such as PARP [24]. The Bax/Bcl-xL ratio is a key factor of mitochondria pathway apoptosis. Proapoptotic proteins, such as Bax, translocate to the mitochondrial membrane and make the mitochondrial outer membrane permeable. Antiapoptotic proteins, such as Bcl-xL, maintain the mitochondrial membrane status by the interactions between proapoptotic proteins [38]. Therefore, the equilibrium between the expression levels of pro- and antiapoptotic proteins is critical for cell survival or death. Presently, apoptosis analysis showed that flavonoids promote apoptosis. Especially, early apoptotic cells increased. Apoptosis induced by flavonoids was also consistent with the observed changes in morphology. Flavonoids caused downregulation of procaspase-3, -6, -8, and -9, which consequently increased the activity of caspase-3. The activation of caspase-3 induced PARP cleavage. In previous studies, the flanovoids genistein and eupatilin induced apoptosis in AGS cells through the activation of caspase-3 and cleaved PARP [39, 40]. Caspase-3 is a very important key of apoptosis and has been recognized as the crucial executioner caspase. Also, cleavage of PARP is a biochemical hallmark of apoptosis. Consistent with this, the flavonoids isolated from Korea *Citrus aurantium* L. induced apoptosis by activating caspase-3 and cleaving PARP.

In conclusion, flavonoids can inhibit the proliferation of AGS cells through both G2/M arrest via the modulation of cell-cycle-related proteins, cyclin B1, cdc 2, and cdc 25c and triggering of apoptosis via upregulation of caspase-3 activity and cleavage of PARP. Although additional investigations are needed to prove the anticancer effects of flavonoids, this study highlights the application potential of flavonoids isolated from Korea *Citrus aurantium* L. in gastric cancer chemotherapy.

## Figures and Tables

**Table 1:** The quantitative value and the retention time of identified flavonoids isolated from *Citrus aurantium* L.

No.	Rt	MS[M+H]+	Compound	Quantity ± SD (mg/kg)
1	16.46	581	Naringin	299.2 ± 0.5
2	18.02	611	Hesperidin	210.3 ± 1.3
3	29.56	595	Poncirin	108.6 ± 1.1
4	41.67	373	Isosinensetin	30.4 ± 0.1
5	42.88	403	Hexamethoxyflavone	15.5 ± 0.05
6	43.40	373	Sinensetin	19.7 ± 0.1
7	44.60	403	Hexamethoxyflavone	23.6 ± 0.07
8	45.91	343	Tetramethyl-o-isoscutellarein	35.6 ± 0.1
9	46.52	403	Nobiletin	200.5 ± 0.1
10	47.50	433	Heptamethoxyflavone	168.9 ± 1.3
11	48.91	419	3-Hydroxynobiletin	28.7 ± 0.1
12	50.78	373	Tangeretin	81.5 ± 0.09
13	52.06	389	Hydroxypentamethoxyflavone	12.8 ± 0.04
14	52.72	403	Hexamethoxyflavone	7.0 ± 0.04
